# Switching polymorph stabilities with impurities provides a thermodynamic route to benzamide form III

**DOI:** 10.1038/s42004-021-00473-7

**Published:** 2021-03-17

**Authors:** Weronika Kras, Andrea Carletta, Riccardo Montis, Rachel A. Sullivan, Aurora J. Cruz-Cabeza

**Affiliations:** 1 Department of Chemical Engineering and Analytical Science, The University of Manchester, Manchester, UK; 2 Chemical Development, Pharmaceutical Technology & Development, Operations, AstraZeneca, Macclesfield, UK; 3 Namur Institute of Structured Matter (NISM), University of Namur, Namur, Belgium

**Keywords:** Thermodynamics, Crystal engineering

## Abstract

Almost 200 years ago, benzamide was reported as polymorphic with two of its forms (II and III) found to be difficult to crystallise. In a recent study, it was shown that benzamide form I can easily convert into benzamide form III using mechanochemistry in the presence of nicotinamide. Here we show, experimentally and computationally, that this transformation is the result of a thermodynamic switch between these two polymorphic forms driven by the formation of solid solutions with small amounts of nicotinamide. The presence of nicotinamide in the crystallisation environment promotes the robust and exclusive crystallisation of the elusive form III. These results represent a promising route to the synthesis and utilisation of elusive polymorphs of pharmaceutical interest.

## Abstract (web summary)

The formation of solid solutions can result in changes of relative stabilities of crystal polymorphs. Here, the elusive form III of benzamide is stabilized through solid solution formation with nicotinamide. It is shown, experimentally and computationally, how such thermodynamic switching allows for consistent and facile crystallization of the otherwise elusive benzamide form III.

## Introduction

Molecular crystals are essential constituents of everyday consumer products such as paints, foods or medicines. The quality, safety and efficacy of such products depend greatly on the properties and structure of the crystals exhibited by their active ingredients. Since most compounds can and will crystallise in various forms (known as polymorphs)^1^, discovering, understanding and controlling polymorphism is a necessity in product development. In the world of pharmaceuticals, over 50% of compounds exhibit polymorphism^2^. Whilst some polymorphs are easy to discover and consistently crystallised, others remain elusive and only appear (or disappear) by chance after decades of research on them^3,4^. The quest for new drugs requires, amongst other things, the synthesis, crystallisation and characterisation of new chemical entities in drug discovery and of the scale up and crystallisation process design in drug development. Along the journey towards a final drug product, it is common to observe the appearance of metastable polymorphs first which eventually transform to other more stable polymorphs. This solid form evolution has often been linked to an increase in chemical purity. When chemical purity is low, compounds can be hard to crystallize or do so in unwanted forms. As the synthetic routes are improved and the compounds are subjected to crystallisation development, their purity increases which may lead to new crystal forms^5^.

Whilst crystallisation is a very selective process (thus widely used as a purification technique), sometimes the tiniest amount of impurities can have a significant impact on the outcome. This is especially true when the impurity has a close molecular similarity with the compound under development. Impurities are well-known to modify crystal habit^6,7^ impact the kinetics of crystallisation^8^ and/or inhibit nucleation, crystal growth, and conversion kinetics between polymorphs^9^ thus promoting or preventing the observation of a particular crystal form. The incorporation of impurities in crystal forms is also known to impact crystal properties such as elasticity or hardness^10^.

Two notorious examples of drugs changing polymorphic forms in the presence of impurities are aspirin and paracetamol. Despite the fact that aspirin has been crystallised in thousands of tons since the 19th century, its second form was only discovered in 2005^11^ when it was crystallised in the presence of impurities (aspirin anhydride)^12–14^. Paracetamol, similarly, can only be crystallised as its form II in the presence of various impurities one of which being metacetamol^15^. Despite the impact that impurities have on polymorphism and crystallisation, there are various molecular mechanisms by which they can act^5^ some of which are not well understood. Pioneering early work by the Weizmann group showed how through preferential adsorption on specific crystal faces, impurities are able to modify crystal habits but also inhibit nucleation and allow for polymorph control^16,17^. More recent work by Liu et al.^18^. has shown how adsorption of metacetamol on form I surfaces in a non crystallographic orientation inhibits its nucleation and growth thus allowing form II to crystallise. In that regard, previous work has mostly focused on using impurities to block the nucleation and growth of stable polymorphs in order to allow for the metastable polymorph to appear. Here we explore a different molecular route of action: rather than blocking the nucleation and growth of the stable form, we explore how other impurities may be able stabilise the metastable polymorph through the formation of solid solutions. Understanding whether or not such routes are possible, and the molecular modes of action would allow us to achieve greater control of polymorphism during crystallisation.

**Fig. 1:**
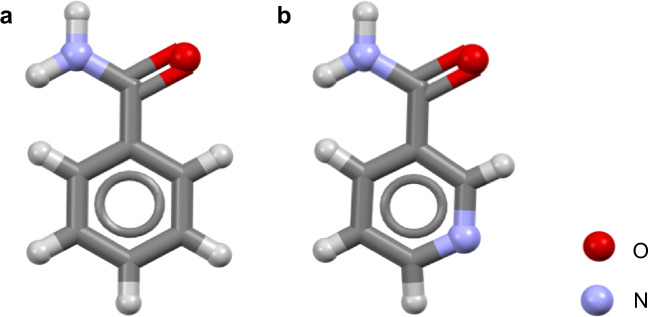
Molecular structures of benzamide (BZM) and nicotinamide (NCM). Molecular structures of (**a**) benzamide and (**b**) nicotinamide.

The polymorphism of benzamide (BZM, Fig. 1a), the first molecular compound ever reported to be polymorphic, was noted by Wöhler and Liebig in 1832^19^ only to be forgotten for over 170 years. Studies on form I (the stable polymorph) abounded and its structure was first reported in 1959^20^. Because of the elusive nature and metastability of the other polymorphs (II and III) and the impossibility to grow large single crystals of good quality suitable for single X-ray diffraction, the solution of the structure of form II and form III had to wait until the advancement of synchrotron radiation^21,22^ and crystal structure solution from powder^23^ methods. More recently, the structure of a metastable and disordered form IV has been proposed^24^. The targeted, robust and exclusive growth of crystals of form III (the form of interest in this contribution) still remains a challenge since this form grows poorly and always concomitantly with form I^25^.

In a recent study by Emmerling and co-workers^26^, a failed attempt to produce 1:1 cocrystals of BZM with its close relative nicotinamide (NCM, Fig. 1b) by ball-mill grinding led to the observation of form III BZM. The solid state BZM form I to form III conversion was consistently observed in a range of BZM:NCM concentrations. The authors mentioned in their conclusions that “it was assumed the nicotinamide molecules were included in the crystal system of benzamide, triggering formation of benzamide III”. If, rather than staying at the crystal surfaces blocking growth, impurities are incorporated in lattice positions within crystal structures, they form what it is known as solid solutions^27^. Inspired by these results, we ventured to investigate this historically important compound further in order to shed light on the driving force of the BZM form I (CSD refcode BZAMID05)^28^ to form III (CSD refcode BZAMID08)^29^ conversion. We reveal, experimentally and computationally, that there is a thermodynamic switch in stability of the BZM polymorphs through the formation of solid-solutions with small amounts of nicotinamide. This switch allows for consistent and robust crystallisation of BZM form III, a polymorph which has remained elusive for almost 200 years.

## Results and discussion

### Mechanochemistry

First, we performed ball-mill liquid assisted grinding (LAG) with ethanol of pure BZM and BZM in the presence of NCM in a range of compositions. LAG of pure BZM affords form I whilst LAG of BZM in the presence of NCM (5-30 mol%) results in BZM form III (Supplementary Results 2.3.1), consistent with Emmerling’s work^26^. At concentrations of NCM above 30 mol%, diffraction peaks corresponding to solid NCM start appearing suggesting that the solid solubility of NCM in BZM form III lies around that value. When ethanol is replaced with isopropanol (IPA), the LAG results do not change. Interestingly, ball mill neat grinding (NG) of pure BZM resulted in pure BZM form III without the need of any NCM at all (Supplementary Results 2.3.2). The observation of form III upon NG of pure BZM may be explained as a crystal size effect^30^. Why small amounts of NCM also induce a BZM form III conversion is less clear since it could potentially be due to a combined size reduction and impurity effect.

### Molecular simulations

**Fig. 2:**
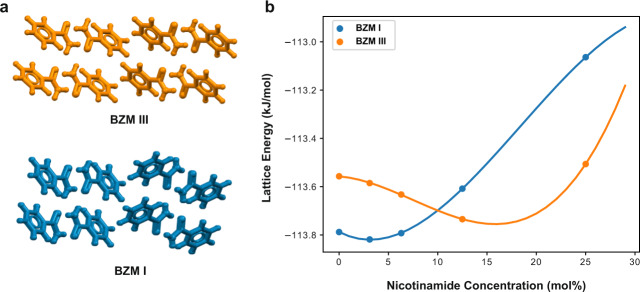
Impact of nicotinamide content on the stability of benzamide forms I and III. **a** Crystal packing of BZM form I (blue) and BZM form III (orange); (**b**) Lattice energies of forms I (blue) and form III (orange) BZM as a function of the NCM concentration. Circles correspond to models for which lattice energies have been calculated (Supplementary Methods 1.3) whilst lines correspond to polynomial fits to the calculated energies - added to aid the eye.

In order to explore the possibility of solid solution formation and its impact on the stability of forms I and III of BZM, we performed lattice energy calculations (PBE-d) for both crystal polymorphs as a function of NCM concentration (Fig. 2). For pure BZM, form I is computed to be more stable than form III by only 0.2 kJ mol^−1^ (Fig. 2b). The small energy difference between the forms is consistent with the fact that forms I and III are very similar in structure (Fig. 2a) and often crystallise concomitantly, with form I known to be the most stable. As the concentration of NCM in the lattice increases, the relative stability of the two polymorphs changes and above 10 mol%, BZM form III becomes more stable than BZM form I. The computed changes in the lattice energies are, however, very subtle. We note that the calculated lattice energies of forms I and III are extremely close (within 0.2-0.4 kJ mol^−1^), with energy differences smaller than the expected accuracies of even the best computational methods available. We also note that computation of positional disorder^31^ in these solid solutions, especially at low NCM concentrations, would have required of the generation of dozens of large supercells (>64 molecules) for the computation of an average static model for disorder. Computing the lattice energies of these supercells at a PBE-d level of theory would be computationally very expensive and was beyond the objective of the present study. Our model relies on a single ordered positional configuration which requires a supercell whereby a single molecule of BZM per cell is substituted by a molecule of NCM. This required the generation of supercells of 4, 8, 16 and 32 molecules (25%, 12.5%, 6.2% and 3.1% NCM respectively, see Supplementary Methods 1.3). The calculations, however, did take into account plausible conformational disorder of NCM. For these, the supercell optimizations were performed twice by considering two conformational configurations of NCM (Supplementary Methods 1.3). One of the configurations (referred to as type B) was always found to form more stable solid solutions than the other (type A) in both crystal structures of forms I and III. Thus, the most stable overall lattice energies for the type B configurations are reported in Fig. 2.

### Solvent-mediated phase transformations and crystallisation experiments

To confirm the revelations of the above simulations, the predicted forms stability changes were assessed experimentally by means of solvent-mediated phase transformation experiments (slurries)^32^. If slurry experiments are given enough time, the lower solubility of the more stable form at each given composition would eventually lead the system to thermodynamic equilibrium and thus the more stable form at such composition.

**Fig. 3:**
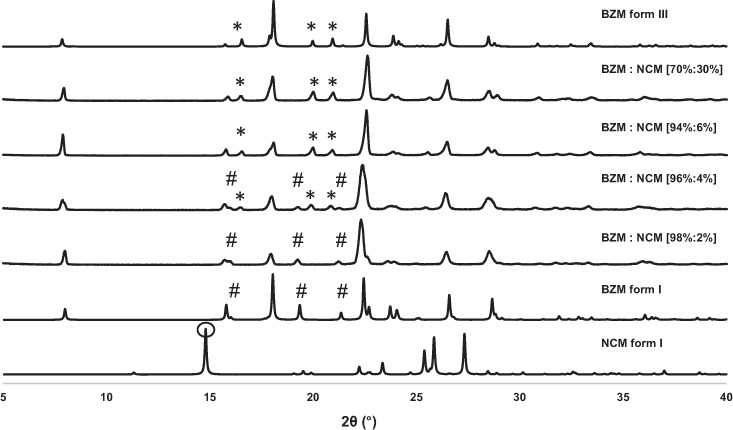
Outcome of the solvent mediated phase transformation experiments. PXRD patterns of slurry products obtained from mixtures of BZM and NCM in IPA at different total BZM:NCM concentrations (in mol% in brackets). Patterns of pure forms are given for comparison (peaks indicated with hashes are typical of BZM form I whilst peaks indicated with asterisk are typical of BZM form III).

Excess solid of BZM form I was stirred in saturated IPA solutions at 25 °C in the presence of various amounts of NCM (Supplementary Results 2.4.2). A 2.5 g of mixed BZM form I with NCM form I solids (at 2, 4, 6, 10 and 20 mol% content of NCM) were added to 5 g of IPA and stirred for one week at which point the solids were characterised. Figure 3 shows the PXRD results for the excess solids of such slurries. Remarkably, the slurry experiments lead to the switch in forms from form I to form III in the presence of NCM. Under these experimental conditions, a total of 4 mol% NCM was required to observe a form I to form III switch in the slurries. When the slurry experiments were repeated in ethanol, the form III conversion was also observed but at slightly higher total concentrations of NCM (7 mol%). These experiments were also performed at higher temperatures, 45 °C, with similar results obtained (Supplementary Results 2.4.1). The amount of impurity present in the slurry had an important effect on the kinetics of the conversion with kinetics accelerating at higher total concentrations of NCM. For example, slurries from ethanol show that when the concentration of NCM is ~10 mol%, the conversion to BZM form III starts after 30 min and completes within 4 h whilst when the NCM concentration is ~20 mol% the conversion to form III completes in just 15 min (Supplementary Results 2.4.1). In all cases, the resulting BZM form III was confirmed to be a solid solution with NCM—as suggested by the absence of diffraction peaks of pure NCM forms, shifts in PXRD peaks and changes in the DSC thermographs. Increasing the concentration of NCM resulted in a linear depression of the melting point of BZM form III from 125.8 °C (10 mol % NCM) to about 121.2 °C (30 mol% NCM) (Supplementary Results 2.4.1). Crash cooling experiments (to 25 °C, 1.2 supersaturations) of BZM in the presence of NCM (~10, 20 and 30 mol%) were also performed in IPA. Consistent with the LAG and the slurry experiments, BZM form III was also obtained via crash cooling in the presence of NCM at all concentrations studied (Supplementary Results 2.5).

**Fig. 4:**
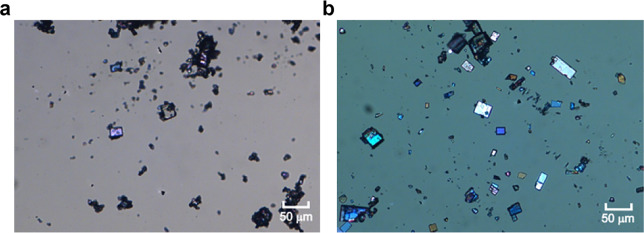
Micrographs of form III benzamide crystals obtained from slurries and crash cooling in IPA. Micrographs of form III BZM crystals obtained from slurries (**a**) and from crash cooling in IPA (**b**) (20 mol% of NCM content). The scalebars are 50 μm.

In the presence of NCM, thus, we are able to consistently and exclusively obtain well-formed form III BZM crystals without the presence of form I crystals, something never achieved before from solution crystallization. This can be achieved both via solvent-mediated phase conversion of form I into form III (slurries) or via crash cooling from solution in the presence of NCM (Fig. 4). We notice that the morphologies of our form III are more equant and similar to those of pure form I and very different to the needles of pure form III BZM^24^. More details about the micrographs can be found in the Supplementary Results 2.7.

### Quantification of nicotinamide incorporation

Until now, we have referred to the amount of NCM content (mol%) with respect to the total amount of solids (BZM + NCM) used in the experiments. In order to estimate the NCM threshold which results in the thermodynamic lattice energy switch (Fig. 2b), we require to characterize the amount of NCM incorporated in forms I and III BZM crystals. This was quantified by retrieving the solids from the slurry experiments in IPA and characterising them with 1H-NMR. The fraction of the NCM incorporated in the crystals over the total NCM fraction in the experiment (in mol%) was calculated to be 0.7 and 0.8 for BZM forms I and III respectively (see Supplementary Results 2.6). Thus, NCM incorporates with very equal efficiency in both lattices. We observe that at around 3 mol% of NCM incorporation in the BZM crystals, form I starts converting to form III, thus the concentration threshold for the stability switch lies around that value. The discrepancy observed between the calculated 10 mol% and the experimental 3 mol% limits can understandably be attributed to the difficulty of simulating energy changes that are so subtle.

### Form I and III similarities

The comparison of the crystal packing of BZM forms I and III reveals a high similarity, with both structures featuring a common 2-D arrangement of centrosymmetric hydrogen-bonded dimers, propagating along the a and b crystallographic directions and connected via a second set of N-H…O hydrogen bonds and other non-directional contacts (Fig. 2a and Supplementary Results 2.2). The only difference between structures occurs along the c direction, where the common 2-D arrangements are packed differently and connected via different sets of weaker contacts. The stability switch of the forms in the presence of NCM is driven by two concomitant but opposite effects: (i) small destabilisation experienced by BZM form I and (ii) the increase of stability of BZM form III upon incorporation of small amounts of NCM. Given the high structural similarity between the two polymorphs, the incorporation of NCM in the crystal lattice is only impacting weak and non-directional contacts subtly. In this case, the “switch” in stability takes place at very small impurity incorporations (3 mol%) because of the close relative lattice energy of the two pure polymorphs.

## Conclusions

In conclusion, we have shown that the relative stability of forms I and III BZM can be inverted by using a selective impurity (NCM) able to form solid solutions. We note that since all compositions studied can be crystallised as forms I or III (and the calculated segregation coefficients of the impurity of both forms were very similar), we have referred to the pure system as well as the solid solutions as polymorphic at each of the given compositions studied. For example, at a 97%:3% BZM:NCM composition, both forms I and III do exist and thus we refer to these forms as polymorphs. The relative stability of the polymorphs changes as a function of composition (thus forms I and III have different relative stabilities in the pure BZM system than in 97%:3% BZM:NCM solid solution). When comparing the pure forms with solid solution forms (i.e., form I pure BZM versus form III 97%:3% BZM:NCM), however, the term polymorph does not further apply. Lattice energy calculations performed on both crystal polymorphs as a function of NCM content suggested a change in the relative stability of the two polymorphs as the concentration of NCM increases. The computed lattice energies of BZM forms I and III as a function of impurity content, however, were extremely similar (within 0.2 kJ mol^−1^) within the accuracy limits of even the best computational models. This highlights the many challenges still ahead for the accurate modelling of polymorphic systems and their solid solutions. Experimentation was used to confirm the predicted polymorph stability switch. Solvent mediated phase transformations showed full conversion from BZM form I to BZM form III starting from concentrations of NCM in the solid lattices of just 3 mol%. The concentration of NCM in the solution was shown to impact the kinetics of the form I to form III conversion, increasing the transformation rates at increasing concentrations of NCM. PXRD, NMR and thermal analysis confirmed the formation of solid solutions. Although impurities are well-known to impact crystal habit, slow nucleation and crystallisation kinetics and impact polymorphism^6–9^, their role in forming solid solutions^33,34^ and the resulting impact on polymorphism has been explored only rarely for organic systems^35–41^. Whilst there has been some experimental work done in this regard^35–41^, to the best of our knowledge, this remains the first study to show the polymorph stability switch experimentally as well as computationally. Most importantly, the fact that only a small amount of impurity is required to switch the forms stabilities is revealing. Thus, this mechanism may be able to explain the appearance and disappearance of some polymorphs^42^ or why in drug research and development early slurry studies give different results to late slurry studies. It may all come down to the presence of inadvertent amounts of impurities and how these are able to switch form stabilities through the formation of solid solutions. Through its understanding, we are now seeking to rationally design such impurities in order to achieve reliable and robust access to elusive or computationally predicted polymorphs through this type of “solid solution thermodynamic switch”. This concept opens a promising route to new polymorphs and their control, which will be of major interest to the pharmaceutical industry.

## Methods

### Materials

Benzamide (BZM) and Nicotinamide (NCM) were purchased from Sigma Aldrich (≥99 %, United Kingdom) and used without further purification. For information about solvents see Supplementary Methods 1.1. The identification of polymorphs methodology is reported in Supplementary Results 2.1.

### Mechanochemical experiments

Ball-mill LAG experiments on BZM/NCM mixtures were performed using the Retsch Mixer Mill MM 200, equipped with 5 ml steel jars and 7 mm diameter stainless steel balls. The experiments were carried out at different BZM/NCM compositions (5.0 wt.%, 10.0 wt.% and 20.0 wt.% and 30.0 wt.%, relative to the total mass of solid). LAG experiments in IPA were carried out using approximately a total mass of 0.3 g of sample and 300.0 µL of IPA, grinding for 60 min at 29 Hz. LAG experiments in ethanol were performed on a total mass of 0.2 g of sample, using 50.0 µL of ethanol, griding for 90 min at 29 Hz. More details are reported in Supplementary Methods 1.2.1.

### Lattice energy calculations

All crystal lattices were subjected to the same optimisation procedure using the software VASP (version 5.3.3)^43–46^. For this, the PBE functiona^47^ with PAW pseudopotentials^48,49^ was used together with the Grimme’s van der Waals corrections^50^. For the planewaves, a kinetic energy cut-off of 520 eV was used. The Brillouin zone was sampled using the Monkhorst–Pack approximation^51^ sing k-points separated by ~0.04 Å (see Supplementary Methods 1.3 Supplementary Table 3). The optimisation cycle performed consisted of two steps: (i) a full geometry optimisation allowing for the unit-cell parameters to change followed by, (ii) a geometry optimisation with the unit cell fixed. Structural relaxations were halted when the calculated force on every atom was less than 0.003 eV Å^−1^. The energy obtained from this process is the electronic energy of the supercell being simulated (*E*^e^_supercell_). The crystal structures of BZM forms I and III were retrieved from the Cambridge Structural Database (CSD refcodes BZAMID05 and BZAMID08 respectively). A number of supercells were then generated for both forms I and III structures (see Supplementary Methods 1.3). A single molecule of BZM in such supercells was then replaced for a molecule of NCM. By generating different supercells with different number of molecules, a single BZM to NCM substitution allows for the simulation of the various solid solution stoichiometries (see Supplementary Methods 1.3). Since NCM has a nitrogen atom in meta position from the amide group, two configurations are possible depending on which of the meta position is occupied by the nitrogen. NCM substitutions were performed in both such configurations A and B. A single molecule of BZM was placed in a 20 Å x 20 Å x 20 Å supercell. The molecule was then allowed to geometry optimise with the cell parameters being fixed. The VASP energy model described above was used and the electronic energy of BZM was calculated (*E*^e^_BZM_). For NCM (configuration A), the same process was repeated and thus the electronic energy of a single NCM molecule was calculated (*E*^e^_NCM_).

The lattice energies of forms I and III with its various levels of NCM incorporated were calculated from the electronic energies of the supercell and the molecules in the gas-phase using Eq. 1.

1 $$E_{latt} = \left( {\frac{{E_{{\it{supercell}}}^e - N_{BZM}E_{BZM}^e - N_{NCM}E_{NCM}^e}}{{N_{{\it{supercell}}}}}} \right)$$

where *N*_supercell_, *N*_BZM_ and *N*_NCM_ are the total number of molecules, number of BZM molecules and number of NCM molecules in the simulation cell, respectively.

This allowed for the calculation of the lattice energy for forms I and III BZM as a function of NCM incorporation in both the configurations A and B. The results are plotted in Supplementary Fig. 1 right. In both forms I and III, configurations B always resulted in lower calculated lattice energies and thus results on configurations B are those given throughout the manuscript. Energies of the A configurations are reported in the Supplementary Methods 1.3.

### Solvent-mediated phase transformation experiments (slurry experiments)

Slurry experiments were carried out in IPA and ethanol and directly characterised by PXRD. Details of the different experiments are described in Supplementary Information (see Supplementary Methods 1.5.1 for slurries in IPA and Supplementary Methods 1.5.2 for slurries in ethanol). Slurries in IPA were carried out on 2.5 g of BZM/NCM mixtures in 5 g of solvent at 25 °C for a week, using the Polar Bear Plus equipment. BZM and NCM were mixed at compositions of 2.0 wt.%, 4.0 wt.%, 6.0 wt.%, 10.0 wt.%, 20 .0 wt.% and 30.0 wt.%, relative to the total mass of solid. Slurries in ethanol were carried out on 5 g of BZM/NCM mixtures in 10 g of solvent at 25 °C and at 45 °C, using a jacketed vessel connected to a thermostated bath. We investigated two range of concentrations (2–5 wt. % and 7-10 wt. %) and 20 wt. %, 30 wt. %, 40 wt. % and 50 wt. %, relative to the total amount of solid. Details about solubility measurements are reported in Supplementary Methods 1.4.

### Crash cooling experiments

BZM/NCM crash cooling experiments were conducted in IPA (supersaturation 1.2 at 25 °C; NCM concentrations: 10 wt.%, 20 wt.% and 30 wt.%), cooling from 40 °C (complete dissolution) to 25 °C (for more details see Supplementary Methods 1.6). After few minutes, block-like crystals appeared in the vessel (see Fig. 4b) which were characterised by PXRD.

### PXRD measurements and DSC measurements

PXRD patterns of the different samples were recorded using a Bruker D2 Phaser diffractometer equipped with a LYNXEYE detector, using a Cu-Kα radiation (*λ* = 1.54184 Å). Intensity data were recorded in the 2θ range of 5-40° (time 0.3 s, increment 0.018°).

DSC measurements were performed using a TA instruments DSC 2500 calorimeter. Approximately 4–5 mg of each sample was placed into an aluminium sealed pan (type TzeroAluminum) and the measurement was carried out under N atmosphere, in the range of 40 °C to 160 °C with a heating ramp of 10 °C min^−1^. The calorimeter was calibrated both for temperature and sensitivity using DSC standards. Data were analysed using the TA TRIOS software. More information is reported in Supplementary Methods 1.7.

### NMR measurements

The incorporation of NCM in the BZM crystal lattice was investigated by ^1^H−NMR, using a 400 MHz NMR spectrometer (Bruker). Details of the sample preparations are described in Supplementary Information (see Supplementary Methods 1.7.3). For each sample obtained from slurry or crash cooling experiments, at least 10 mg of crystals were dissolved in 550 μL of acetone-d_6_ and the NCM/BZM ratios were calculated on the basis of the integrated areas of the peaks.

## Supplementary information

Supplementary Information

Description of Additional Supplementary Files

Supplementary Data 1

## Data Availability

PXRD and thermal analysis data are included in this published article (and its supplementary information files). NMR spectra are available on request. Geometry optimised models for the solid solutions are available as Supplementary Data 1.
