# Supplementary material for: Switching polymorph stabilities with impurities provides a thermodynamic route to benzamide form III
Source: Commun Chem. 2021 Mar 17;4:38. doi: 10.1038/s42004-021-00473-7 (PMC9814557; doi:10.1038/s42004-021-00473-7)
Supplement: Supplementary file 1 — Supplementary Information [file 42004_2021_473_MOESM1_ESM.pdf]

Switching Polymorph Stabilities with Impurities Provides A Thermodynamic Route to Benzamide Form III.

Weronika Kras,[a, b] Andrea Carletta, [c] Riccardo Montis, [a] Rachel A. Sullivan,[b] Aurora J. Cruz-Cabeza*[a,b]

[a]Department of Chemical Engineering and Analytical Science, The University of Manchester, Oxford Road, Manchester, M13 0PL, UK, E-mail: aurora.cruzcabeza@manchester.ac.uk

[b]Chemical Development, Pharmaceutical Technology & Development, Operations, AstraZeneca, Macclesfield. UK

[c]Namur Institute of Structured Matter (NISM), University of Namur, 61 rue de Bruxelles, B-5000 Namur, Belgium

Table of Contents

Supplementary Methods 2
1.1 Materials 2
1.2 Grinding 2
1.2.1 Liquid Assisted Grinding (LAG) 2
1.2.2 Neat grinding 2
1.3 Molecular Simulations 3
1.4 Solubility Measurement 5
1.5 Slurry Experiments 5
1.5.1 Isopropanol based slurries 5
1.5.2 Ethanol based slurries 6
1.6 Crash Cooling Experiments 6
1.7 Characterisation Techniques 7
1.7.1 PXRD measurements 7
1.7.2 DSC measurements 7
1.7.3 NMR measurements 7
1.7.4 Optical Microscopy 7
Supplementary Results 8
2.1 Identification of polymorphs 8
2.2 Crystal packing comparison of Form I and Form III 8
2.3 Grinding experiments 9
2.3.1 LAG 9
2.3.2 Neat grinding 11
2.4 Slurry Experiments 12
2.4.1 Slurries in ethanol 12
2.4.2 Slurries in isopropanol 15
2.5 Crash Cooling 16
2.6 Incorporation levels/ Segregation Coefficients 17
2.7 Optical Microscopy images 18
Supplementary References 19

Supplementary Methods

1.1 Materials

Benzamide (BZM) and Nicotinamide (NCM) were purchased from Sigma Aldrich (≥ 99 %, United Kingdom) and used without further purification. Deuterated acetone (acetone D6, 99.8%) was purchased from VWR International Ltd. and used without any further purification for NMR analysis. Isopropanol and ethanol were obtained from Fisher Scientific (>98%, United Kingdom).

1.2 Grinding

1.2.1 Liquid Assisted Grinding (LAG)

LAG experiments in isopropanol were carried out using the Retsch Mixer Mill MM 200. Approximately a total mass of 0.3 g of sample, 300.0 µL of isopropanol and 7 mm diameter stainless steel ball were loaded to a 5 ml steel jar. The contents of additive relative to the total mass of solid (BZM + NCM), were 5.0 wt.%, 10.0 wt.%, 20.0 wt.%, 30.0 wt.% and 40 wt.%. After addition of 300.0 µL of isopropanol to each jar, grinding for 1 h at 29 Hz was conducted.

LAG experiments in ethanol were carried out using the Retsch Mixer Mill MM 200. Approximately a total mass of 0.2g of sample, 50.0 µL of ethanol and 7 mm diameter stainless steel ball were loaded to a 5 ml steel jar. The contents of additive relative to the total mass of solid (BZM + NCM) were 5.0 wt.%, 10.0 wt.%, 20.0 wt.% and 30.0 wt.% (corresponding to 5.0, 9.9, 19.9 and 29.8 mol%, respectively). After addition of 50.0 µL of ethanol to each jar, grinding for 90 min at 29 Hz was conducted. Supplementary Table 1 details the compositions used in both LAG experiments.

Supplementary Table 1. NCM and BZM quantities used in LAG.

Solvent	Wt.%[a]NCM	Mass NCM (g)	Wt.%[a] BZM	Mass BZM (g)
**Isopropanol**	5	0.015	95	0.285
	10	0.030	90	0.270
	20	0.060	80	0.240
	30	0.090	70	0.210
	40	0.120	60	0.180
**Ethanol**	5	0.010	95	0.190
	10	0.020	90	0.180
	20	0.040	80	0.1600
	30	0.060	70	0.140

[a] wt.% with respect to the total solid load used

1.2.2 Neat grinding

For ball-mill neat grinding Retsch Mixer Mill MM 400 was used with 5 ml steel jar and 7 mm diameter stainless steel ball. In this case no additive nor solvent were used. Total mass of 0.3 g of BZM was ground for 1 hr at 29 Hz.

1.3 Molecular Simulations

Crystal structure models

The crystal structures of benzamide forms I and III were retrieved from the Cambridge Structural Database (CSD refcodes BZAMID05[1] and BZAMID08[2] respectively). Basic crystallographic information for these two forms is provided in Supplementary Table 2.

Supplementary Table 2. Crystallographic information for forms I and III benzamide.

	**Form I**	**Form III**
REFCODE	BZAMID05	BZAMID08
T (K)	173	283-303
SpaceGroup	$P2_1/c$	$P2_1/c$
Z'	1	1
a, b, c (Å)	5.57, 5.04, 21.70	5.06, 5.51, 22.96
$\alpha, \beta, \gamma (°)$	90.0, 90.4, 90.0	90.0, 101.3, 90.0

A number of supercells were then generated for both forms I and III structures (Supplementary Table 2). A single molecule of BZM in such supercells was then replaced for a molecule of NCM. By generating different supercells with different number of molecules, a single BZM to NCM substitution allows for the simulation of the various solid solution stoichiometries (Supplementary Table 2). Since NCM has a nitrogen atom in meta position from the amide group, two configurations are possible depending on which of the meta position is occupied by the nitrogen (configuration A and B, Supplementary Figure 1 left). NCM substitutions were performed in both such configurations A and B.

Geometry optimisations

All crystal lattices were subjected to the same optimisation procedure using the software VASP (version 5.3.3 [3–6]). For this, the PBE functional[7] with PAW pseudopotentials [8,9] was used together with the Grimme's van der Waals corrections.[10] For the planewaves, a kinetic energy cut-off of 520 eV was used. The Brillouin zone was sampled using the Monkhorst–Pack approximation[11] using k-points separated by approximately 0.05 Å (see Supplementary Table 3). For cell lengths of 5-6 Å four K-points were used in such direction, for cell lengths of ~9-11 Å two K-points and for cell lengths of > 20 Å one K-point.

The optimisation cycle performed consisted of two steps: i) a full geometry optimisation allowing for the unit-cell parameters to change followed by, ii) a geometry optimisation with the unit cell fixed. Structural relaxations were halted when the calculated force on every atom was less than 0.003 eV $Å^{-1}$. The energy obtained from this process is the electronic energy of the supercell being simulated ($E^{e}_{supercell}$).

Supplementary Table 3. Supercells generated for forms I and III and the NCM substitutions.

Form	Mol% NCM	Supercell Construction	Input Cell	#Molecules Cell	#Molecules BZM	#Molecules NCM	K-points
I (BZAMID05)	0	1 x 1 x 1	5.6 x 5.0 x 21.7	4	4	0	4 x 4 x 1
	3.1	4 x 2 x 1	22.4 x 10.0 x 21.7	32	31	1	1 x 2 x 1
	6.2	2 x 2 x 1	11.2 x 10.0 x 21.7	16	15	1	2 x 2 x 1
	12.5	1 x 2 x 1	5.6 x 10.0 x 21.7	8	7	1	4 x 2 x 1
	25	1 x 1 x 1	5.6 x 5.0 x 21.7	4	3	1	4 x 4 x 1
III (BZAMID08)	0	1 x 1 x 1	5.0 x 5.5 x 22.9	4	4	0	4 x 4 x 1
	3.1	4 x 2 x 1	20.0 x 11.0 x 22.9	32	31	1	1 x 2 x 1
	6.2	2 x 2 x 1	10.0 x 11.0 x 22.9	16	15	1	2 x 2 x 1
	12.5	1 x 2 x 1	5.0 x 11.0 x 22.9	8	7	1	4 x 2 x 1
	25	1 x 1 x 1	5.0 x 5.5 x 22.9	4	3	1	4 x 4 x 1

Similar DFT methodologies were used in the literature for the computational study of BZM polymorphs.[12,13]

Calculation of gas-phase energy of BZM and NCM

A single molecule of BZM was placed in a 20 Å x 20 Å x 20 Å supercell. The molecule was then allowed to geometry optimise freely (the cell parameters being fixed). The VASP energy model described above was used and the electronic energy of BZM was calculated (E^{e}_{BZM}).

For NCM (configuration A), the same process was repeated and thus the electronic energy of a single NCM molecule was calculated (E^{e}_{NCM}).

Calculation of lattice energies

The lattice energies of forms I and III with its various levels of NCM incorporated were calculated from the electronic energies of the supercell and the molecules in the gas-phase using the equation below:

$$E_{latt} = \left(\frac{E^{e}_{supercell} - N_{BZM}E^{e}_{BZM} - N_{NCM}E^{e}_{NCM}}{N_{supercell}} \right)$$

where $N_{supercell}$, N_{BZM} and N_{NCM} are the total number of molecules, number of BZM molecules and number of NCM molecules in the simulation cell.

This allowed for the calculation of the lattice energy for forms I and III BZM as a function of NCM incorporation in both the configurations A and B. The results are plotted in Supplementary Figure 1 right. In both forms I and III, configurations B always resulted in lower calculated lattice energies and thus results on configurations B are given in the main manuscript.

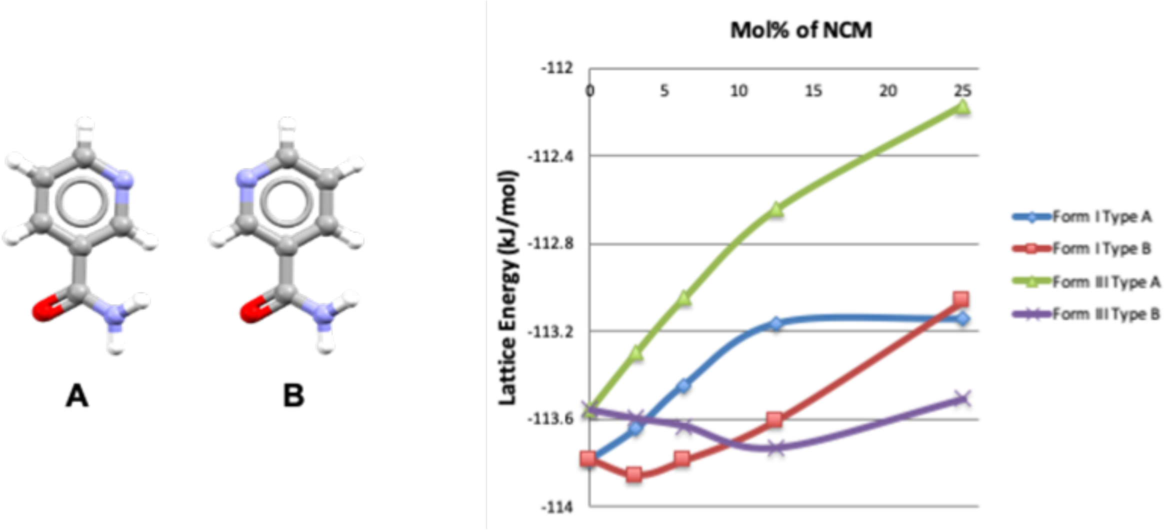

Supplementary Figure 1. (left) Configurations A and B of NCM. (right) Lattice energy as a function of NCM concentration in forms I and III BZM with type A and B configurations.

1.4 Solubility Measurement

Solubility measurements at 25°C of mixtures of BZM with NCM (concentrations: 10 wt.%, 20 wt.% and 30 wt.%) in isopropanol were determined by gravimetric methods. Suspensions of the different mixtures were equilibrated under stirring for 1 week. For each concentration, aliquots of approximately 0.8 mL were transferred into 5 pre-weighted vials using a disposable syringe with a filter (0.22 µm PES filter). The mass of the saturated solution was then recorded using an analytical balance (HM-202, A&D Company, Limited with a precision of ± 0.0001 g) and all vials were placed in vacuum oven for 48 h to allow for the evaporation of the solvent. The amount of dried solid in each vial was recorded and then the average solubility for each NCM concentration could be calculated.

1.5 Slurry Experiments

1.5.1 Isopropanol based slurries

All slurry experiments were conducted using a total amount of solid mixture of 2.5 g and 5 g of Isopropanol. The contents of NCM relative to the total mass of solid were 2.0 wt.%, 4.0 wt.%, 6.0 wt.%, 10.0 wt.%, 20 .0 wt.% and 30.0 wt.% (corresponding to 2.0, 4.0, 6.0, 9.9, 19.9 and 29.8 mol%, respectively). All samples underwent slurrying for a week using Polar Bear Plus at 25°C. The solutions were stirred continuously for 1 week by magnetic stirrers to ensure that solid-liquid equilibrium is achieved. After a week the slurries were filtered under vacuum and the resulting powder was immediately characterised via PXRD.

1.5.2 Ethanol based slurries

Slurries were performed in a jacketed vessel at 25 °C and at 45°C in 10 g of ethanol using a total load of 5 g of solid. The different amount of NCM tested were of 2-5 %, 7-10 %, 20 %, 30 %, 40 % and 50 % (expressed as wt% with respect to total solid load of 5 g). Supplementary Table 4 contains all initial compositions of slurries prepared in isopropanol and ethanol.

Supplementary Table 4. Concentrations of BZM and NCM in the investigated slurries.

**Isopropanol (g)**	5				
**Total solid (g)**	2.5				
**Total Amount of Solution (g)**	7.5				
**BZM wt.%[a]**	**NCM wt.%[a]**	**Mass BZM (g)**	**Mass NCM (g)**	**BZM wt.% in solution[b]**	**NCM wt.% in solution[b]**
98.000	2.000	2.450	0.050	32.667	0.667
96.000	4.000	2.400	0.100	32.000	1.333
94.000	6.000	2.350	0.150	31.333	2.000
90.000	10.000	2.250	0.250	30.000	3.333
80.000	20.000	2.000	0.500	26.667	6.667
70.000	30.000	1.750	0.750	23.333	10.000
**Ethanol (g)**	10				
**Total solid (g)**	5				
**Total amount of solution (g)**	15				
**BZM wt.%[a]**	**NCM wt.%[a]**	**Mass BZM (g)**	**Mass NCM(g)**	**BZM wt.% in solution**	**NCM wt.% in solution**
98.000	2.000	4.900	0.100	32.667	0.667
97.000	3.000	4.850	0.150	32.333	1.000
96.000	4.000	4.800	0.200	32.000	1.333
95.000	5.000	4.750	0.250	31.667	1.667
93.000	7.000	4.650	0.350	31.000	2.333
92.000	8.000	4.600	0.400	30.667	2.667
91.000	9.000	4.550	0.450	30.333	3.000
90.000	10.000	4.500	0.500	30.000	3.333
80.000	20.000	4.000	1.000	26.667	6.667
70.000	30.000	3.500	1.500	23.333	10.000
60.000	40.000	3.000	2.000	20.000	13.333
50.000	50.000	2.500	2.500	16.667	16.667

[a] – wt.% with respect to total solid used

[b] – wt.% with respect to the total amount of solution, including solvent

1.6 Crash Cooling Experiments

Crash cooling experiments were conducted in isopropanol at 1.2 supersaturation for samples with NCM concentrations of 10 wt.%, 20 wt.% and 30 wt. % (where these percentages are with respect to the total solid needed to achieve the set supersaturation value – more details in Supplementary Table 5). For each sample, a stirred supersaturated solution was heated up to approximately 40 °C until complete

dissolution. The solution was then quickly cooled down to 25 °C using a LAUDA ECO RE 415 water bath. Once the solution was cooled down it took few minutes for the crystals to nucleate and grow. The resulting crystalline material was then filtered and characterised by PXRD.

Supplementary Table 5. Compositions of crash cooled solutions that were investigated.

**Isopropanol (g)**			**20**		
**NCM wt.% [a]**	Solubility of the solid solution g/gIPA	Mass of NCM (g)	Mass of BZM (g)	BZM in solution (wt.%)[b]	NCM in solution (wt.%)[b]
**10**	0.110	0.264	2.376	10.495	1.166
**20**	0.140	0.670	2.679	11.474	2.869
**30**	0.143	1.027	2.396	10.229	4.384

[a] – wt.% with respect to the total solid used

[b] - wt.% with respect to both the total solid and solvent used

1.7 Characterisation Techniques

1.7.1 PXRD measurements

PXRD patterns for the different samples were recorded using a Bruker D2 Phaser diffractometer equipped with a LYNXEYE detector, using a Cu-Kα radiation (λ= 1.54 Å). Intensity data were recorded in the 2θ range of 5-40° (time 0.3 s, increment 0. 018°).

1.7.2 DSC measurements

DSC measurements were performed using a TA instruments DSC 2500 calorimeter. Approximately 4 - 5 mg of each sample was placed into an aluminium sealed pan (type TzeroAluminum) and the measurement was carried out under N atmosphere, in the range of 40 °C to 160 °C with a heating ramp of 10 °C min^{-1}. The calorimeter was calibrated both for temperature and sensitivity using DSC standards. Data were analysed using the TA TRIOS software.

1.7.3 NMR measurements

NMR measurements were undertaken to estimate the incorporation of NCM in BZM crystal lattice during slurry and crash cooling experiments. The NCM content in such samples was detected by 1 H−NMR measurements (128 scans per sample) carried out using a 400 MHz NMR spectrometer (Bruker). Deuterated acetone was used in the NMR samples to dissolve the crystals used for analysis. The proton NMR scans were recorded and analysed using MestReNova version 14.1.0−24037[14]. The accuracy of the method was validated using a solution containing a molar ratio of BZM to NCM being equal to 2.375, which was correctly quantified to contain a molar ratio of BZM to NCM corresponding to 2.37.

1.7.4 Optical Microscopy

Samples from slurries and crash cooling were analysed using Zeiss Axioplan 2 microscope and images were obtained using the INFINITY Analyse and Capture Software version 6.5.6.

Supplementary Results

2.1 Identification of polymorphs

BZM

The crystalline structures of BZM powders/crystals were identified by comparing the experimental PXRD patterns with the calculated patterns from the single crystal structures obtained from CSD: BZAMID05 (form I)[1] and BZAMID08 (from III)[2].

NCM

The crystalline structure of commercial NCM powder was confirmed by comparing the experimental PXRD pattern with the calculated patterns from the single crystal structures obtained from CSD: NICOAM05 (form I)[15].

2.2 Crystal packing comparison of Form I and Form III

A crystal structure comparison of BZM I and III has been carried out using the XPac procedure[16] using the whole molecule of BZM I as the common ordered set of points (COSP). Results of the comparison, including the dissimilarity index (χ), are reported in Supplementary Table 6. Supplementary Figure 2 shows the structural similarity and a description of the crystal packing of BZM I and III.

Supplementary Table 6. Results of the crystal packing comparison (XPac). Lattice vectors, angles and dissimilarity index (χ) of the 2-D similarity.

Polymorph1[a]	Corresponding lattice vector 1 (Å)	Corresponding lattice vector 2 (Å)	Angle (˚)	χ
BZM I	[010]: 5.046	[100]: 5.607	90	6.6
BZM III	[100]: 5.055	[010]: 5.514	90	-

[a] Ref codes: BZAMID05 (form I)[1] and BZAMID08 (from III)[2].

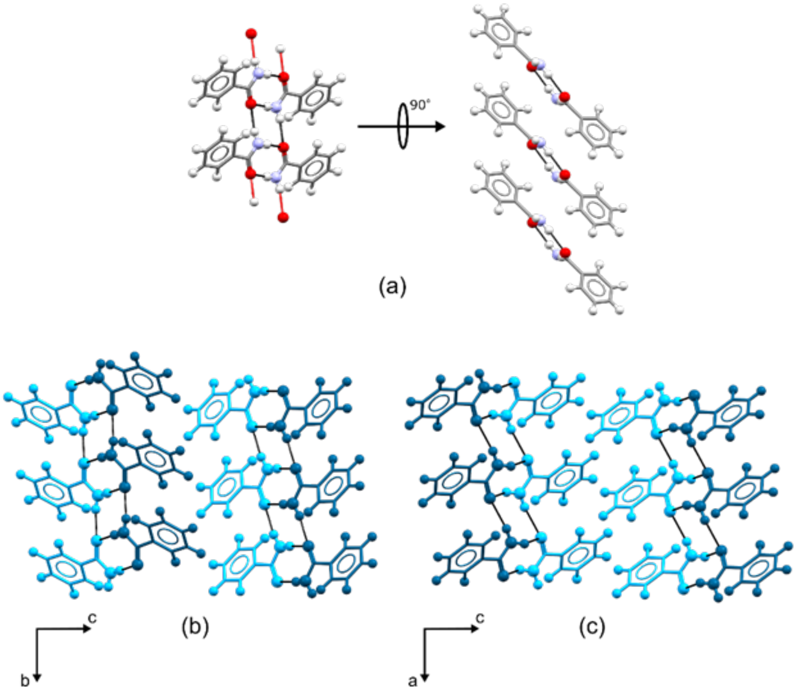

Supplementary Figure 2. Crystal packing of BZM I and BZM III. a) Structural similarity: common 2-D molecular arrangement. b) crystal packing of BZM I (refcode BZAMID05[1]). c) Crystal packing of BZM III (refcode BZAMID08[2]). Molecules are colour coded according to the orientation of the ring-amide functional group vector: toward the viewer (light blue), opposite to the viewer (blue).

2.3 Grinding experiments

2.3.1 LAG

Supplementary Figures 3 and 4 show PXRD patterns of the BZM-NCM system prepared by LAG at NCM concentrations in the ranges of 5-30 (wt.%) and 5-40% (wt.%) with ethanol and isopropanol, respectively. In all the cases, powder patterns show a good fit with that of BZM form III, calculated from the single-crystal structure (BZAMID08[2]). At NCM concentration of 40%, extra peaks corresponding to NCM form I appear (blue arrows) in the powder pattern, suggesting that 30-40% represents the solubility limit of NCM into BZM.

Supplementary Figures 3 and 4 also show linear shifts of the PXRD peaks to higher values of 2θ as the concentration of NCM increases (see also Supplementary Figure 5). This suggests a decrease of the planar spacing as BZM is partially replaced by NCM, resulting in a solid solution.

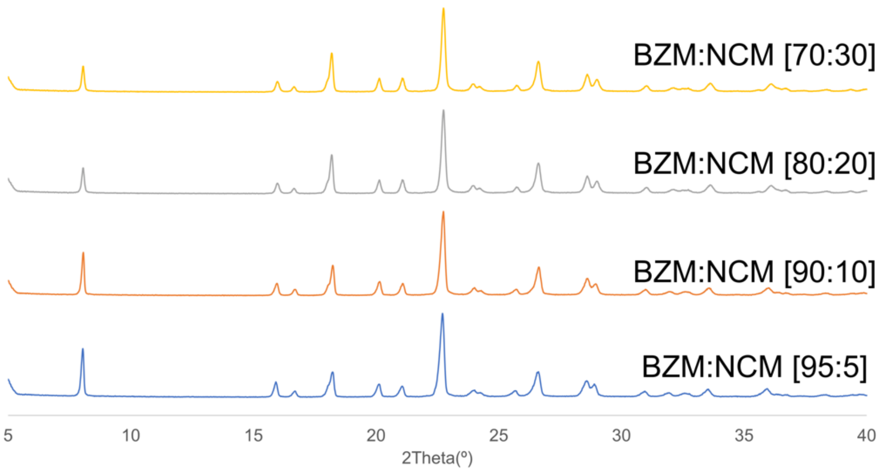

Supplementary Figure 3. PXRD patterns of LAG products containing various concentrations of NCM (LAG with ethanol)

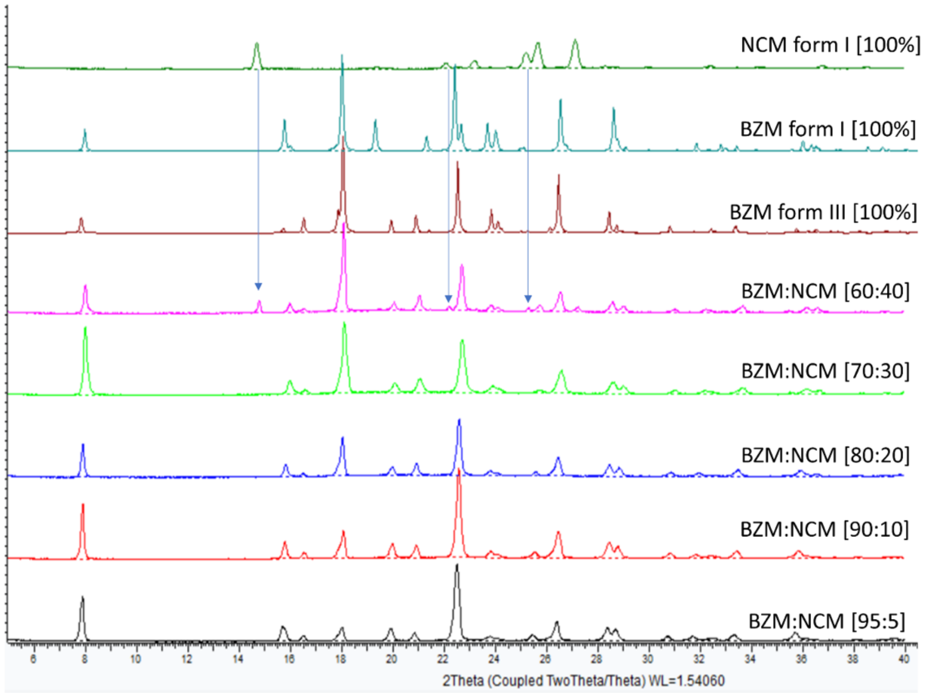

Supplementary Figure 4. PXRD patterns of LAG products containing various concentrations of NCM (LAG with isopropanol)

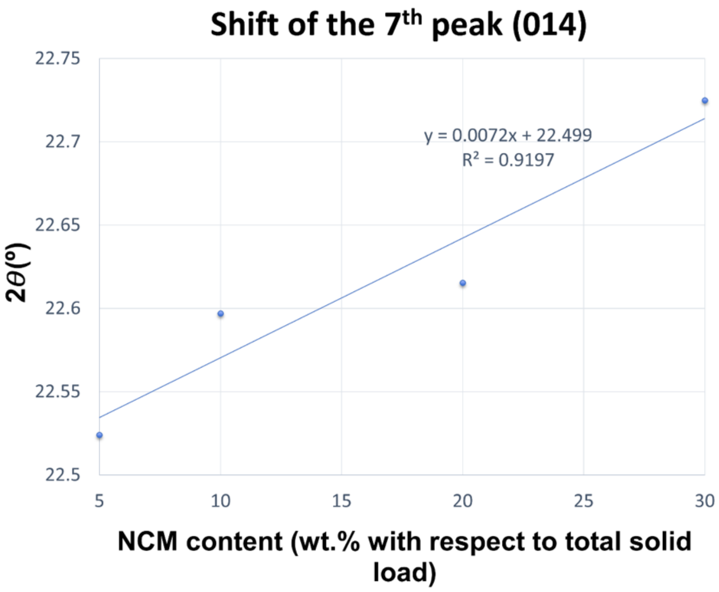

Supplementary Figure 5. The most linear peak shift identified from PXRD patterns obtained from LAG samples with isopropanol.

2.3.2 Neat grinding

Supplementary Figure 6 shows a comparison of the PXRD patterns of BZM obtained from neat grinding with BZM form I and BZM form III patterns calculated from the single crystal-structures (BZAMID05[1] and BZAMID08[2], respectively). The results clearly show that neat grinding of pure BZM promotes the conversion of BZM form I to BZM form III.

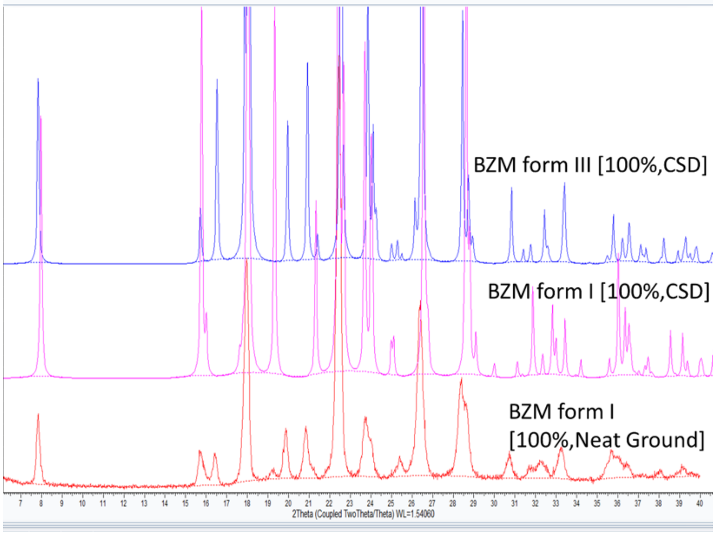

Supplementary Figure 6. The PXRD pattern of BZM exposed to neat grinding, and its comparison to BZM form I and BZM form III patterns obtained from CSD.

2.4 Slurry Experiments

2.4.1 Slurries in ethanol

Supplementary Figures 7 and 8 show the PXRD patterns of samples obtained from slurries of BZM form I in the presence of NCM at 25°C and 45°C, respectively. The results show that the conversion to BZM form III starts at NCM concentration of approximately 7 wt.%.

In order to determine the kinetics of the polymorphic conversion of BZM form I to BZM form III, slurry experiments of the mixture BZM /NCM were conducted in ethanol at different concentrations of NCM and the suspensions were characterized by PXRD. Supplementary Figures 9 and 10 show PXRD patterns for samples of BZM in the presence of NCM at concentrations 10% (wt.%) and 20 (wt.%), respectively

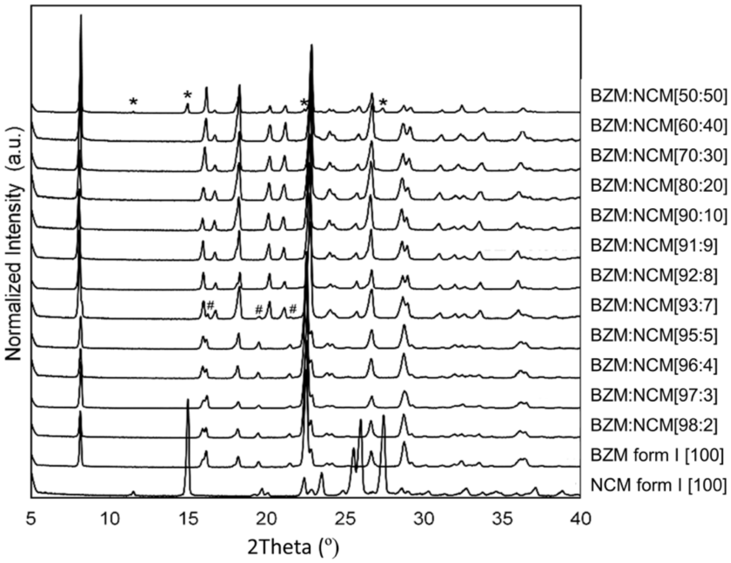

Supplementary Figure 7. Powder diffraction patterns of filtered powders from slurry experiments in ethanol (25 °C). The I to III BZM polymorphic conversion starts taking place in the presence of 7% of NCM. Hashes indicate residual peaks of form I. Asterisks indicate residual peaks of NCM.

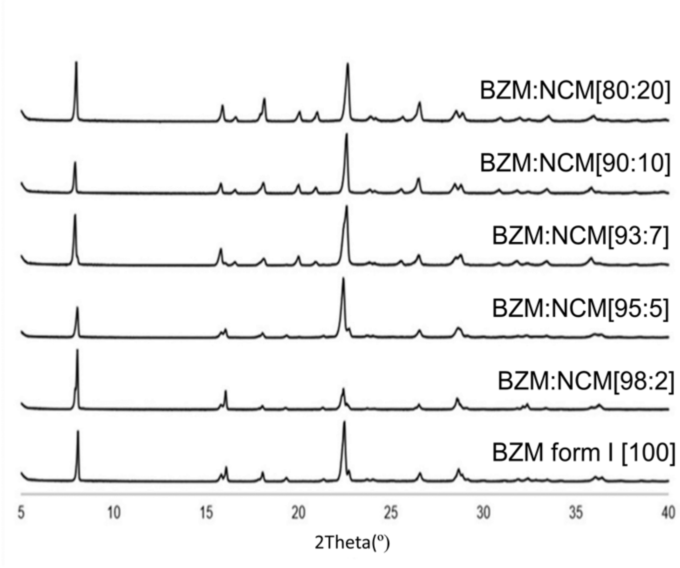

Supplementary Figure 8. Powder diffraction patterns of filtered powders from slurry experiments in ethanol (45 °C). The I to III BZM polymorphic conversion also starts taking place in the presence of 7% of NCM.

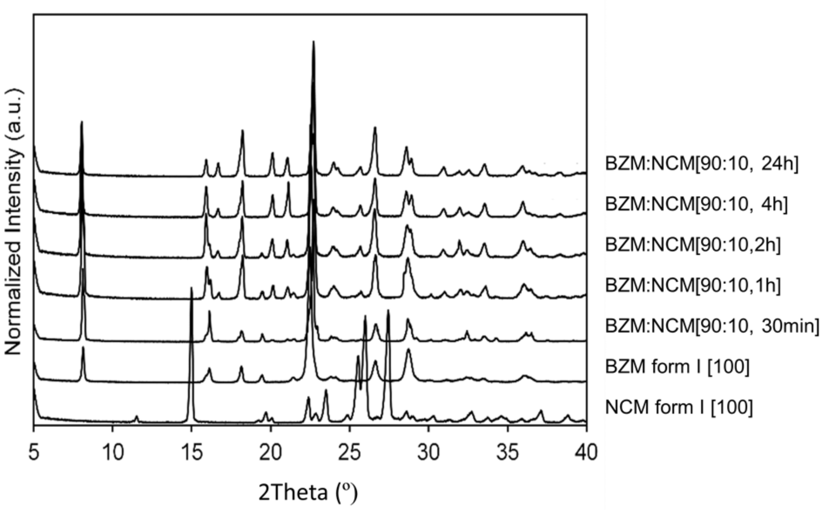

Supplementary Figure 9. The kinetics of the polymorphic conversion of BZM in the presence of NCM (10 wt.%) in ethanol slurries.

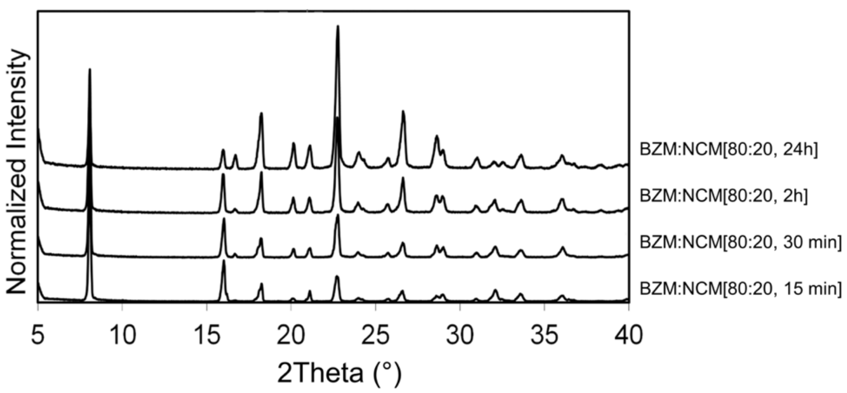

Supplementary Figure 10. Evolution over time of the BZM I to III conversion in the presence of 20% of NCM in ethanol slurries.

DSC thermograms of crystals obtained from ethanol slurries at various NCM doping levels are shown in Supplementary Figure 11. Single endothermic event can be observed, which is a typical behaviour for solid solution formation. Furthermore, as the doping level of NCM increases, melting point depression occurs, also characteristic of solid solutions. The latter along with the enthalpy of fusion changes are shown in Supplementary Figure 12. For comparison, Supplementary Figure 13 shows the DSC thermographs of manually prepared physical mixtures of BZM and NCM. Two peaks are clearly observed, further confirming a solid solution formation when BZM and NCM system undergoes slurrying.

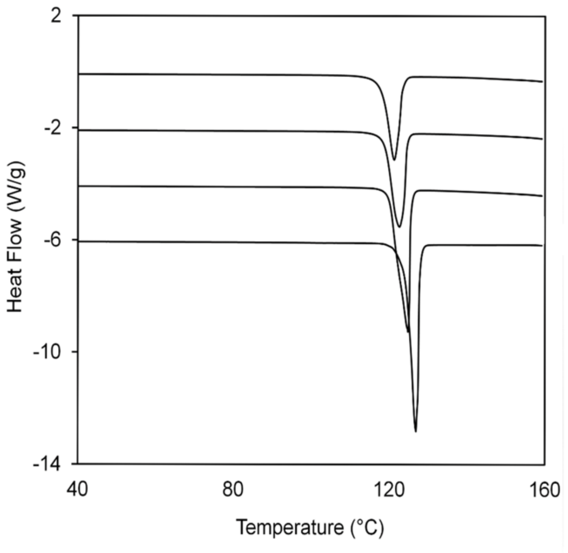

Supplementary Figure 11. DSC thermographs of products from ethanol slurries.

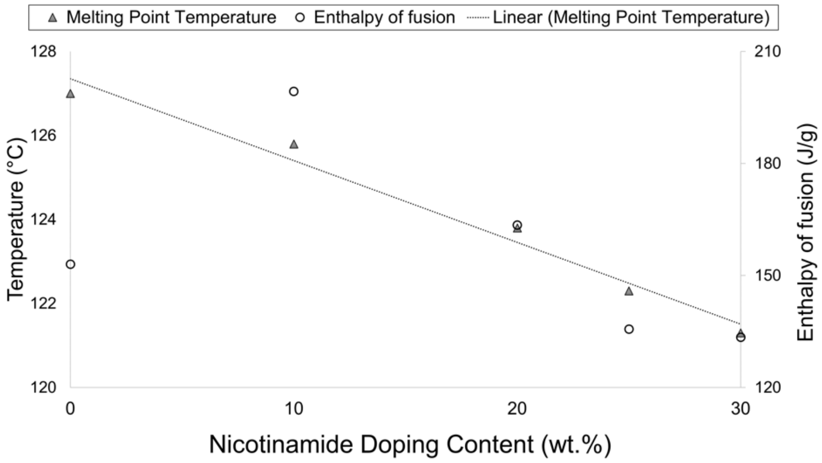

Supplementary Figure 12. Variation of the melting point temperature and the enthalpy of fusion with NCM doping level.

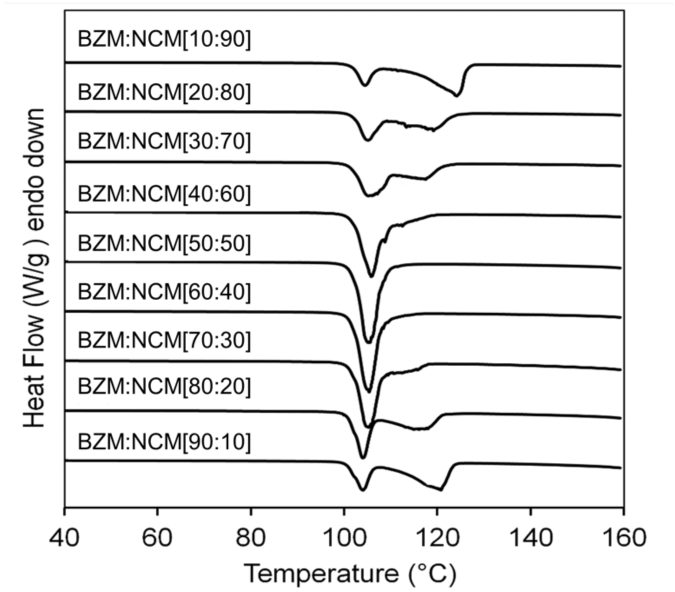

Supplementary Figure 13. DSC thermographs of physical mixtures of BZM with NCM. Eutectic can be seen at NCM concentration of 40%.

2.4.2 Slurries in isopropanol

In order to ascertain whether a different solvent also promotes the conversion of BZM form I to BZM form III in the presence of NCM, slurry experiments in isopropanol were carried out. Supplementary Figure 14 shows the PXRD patterns of samples obtained by slurrying BZM form I in the presence of NCM concentrations of 2 - 30% (wt.%). The results show that the conversion to BZM form III starts at NCM doping level of around 4% (wt.%).

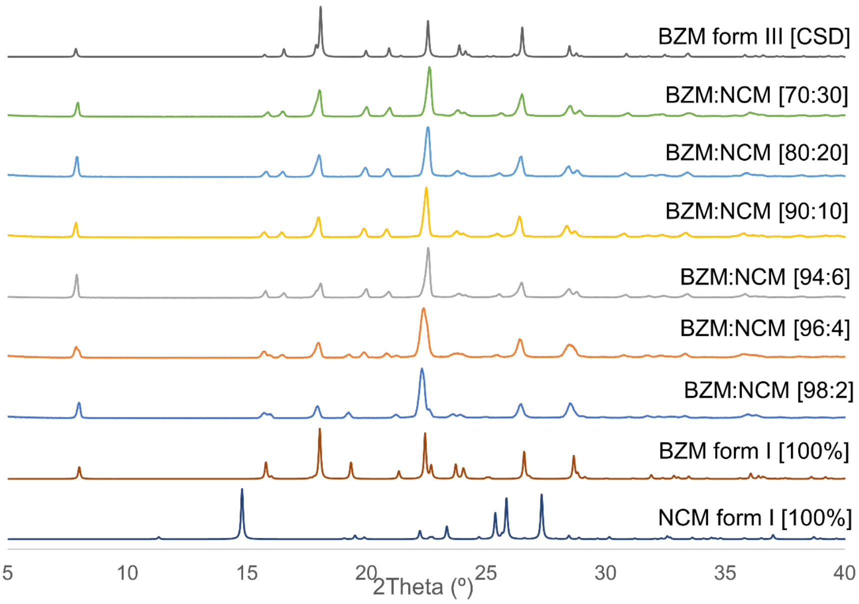

Supplementary Figure 14. PXRD patterns of slurry products obtained from mixtures of BZM and NCM in isopropanol at different wt.% doping levels.

2.5 Crash Cooling

Supplementary Figure 15 shows PXRD patterns of samples obtained from crash cooled solutions of BZM in the presence of 10%, 20% and 30 % of NCM (wt.%). In all cases the resulting PXRD pattern shows a good fit with that of BZM form III.

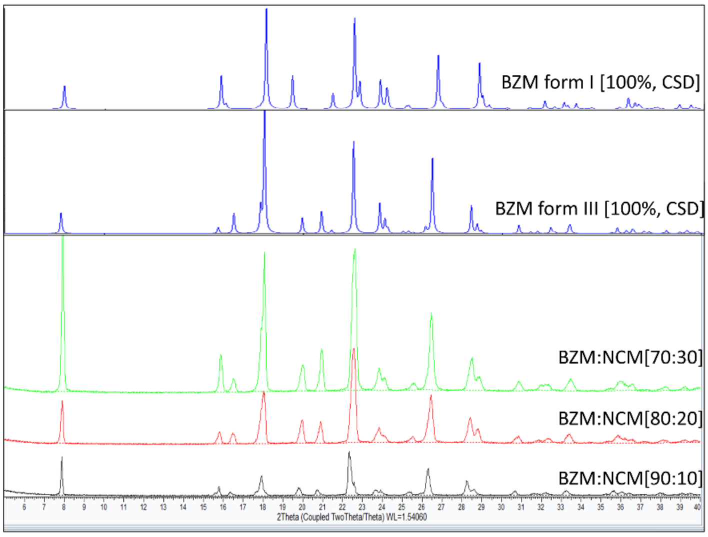

Supplementary Figure 15. PXRD patterns of crash cooling samples obtained from mixtures of BZM and NCM in isopropanol.

2.6 Incorporation levels/ Segregation Coefficients

Supplementary Figure 16 shows the differences between the incorporation of NCM in the crystal lattice BZM during slurry and crash cooling experiments in isopropanol. Higher incorporations of NCM occur during slurry experiments, corresponding to a higher segregation coefficient, which is indicated by the slope of the trendline.

Supplementary Figure 17 demonstrates the differences between the incorporation of NCM in BZM form III and BZM form I at slurring conditions (isopropanol slurries). It was observed that more NCM incorporates in BZM form III lattice than in form I lattice at equilibrium conditions. This is also demonstrated by the estimated segregation coefficients, being 0.8 for NCM in BZM form III and 0.7 for NCM in BZM form I, which are however very close.

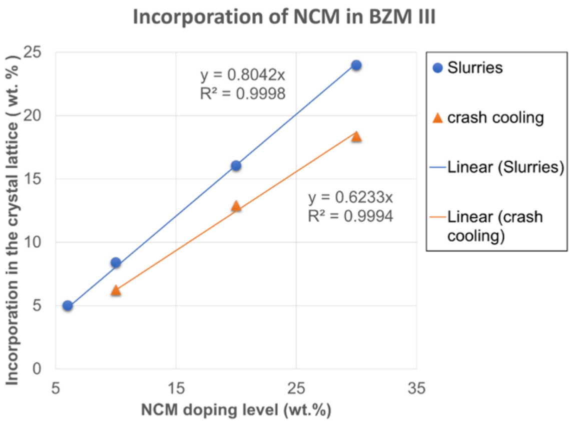

Supplementary Figure 16. The comparison between the incorporation of NCM in BZM form III at slurry and at crash cooling conditions.

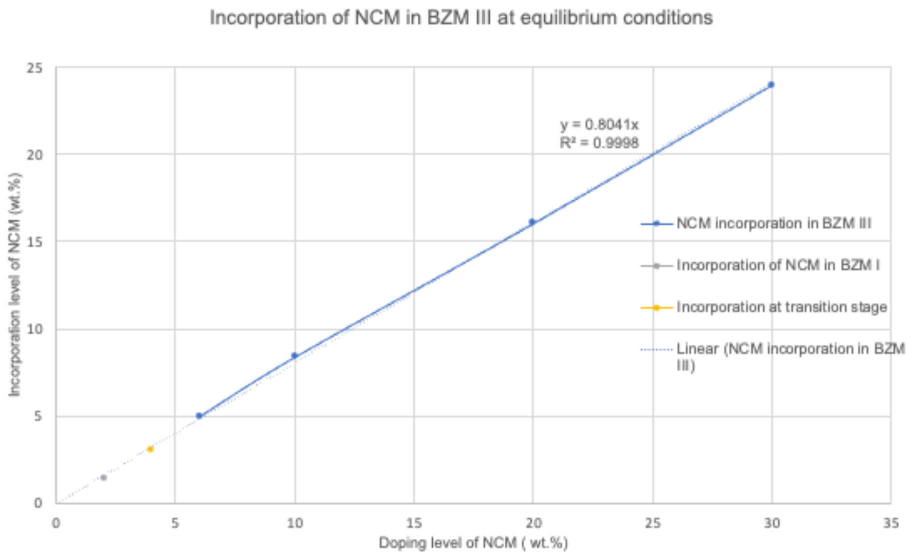

Supplementary Figure 17. The differences between the incorporation of NCM in BZM form III and BZM form I at slurrying conditions.

2.7 Optical Microscopy images

Supplementary Figures 18 and 19 show microscopy images of crystallites obtained from slurries in isopropanol and crash cooling crystallisation, respectively. The initial solution concentration for both samples had 20 wt.% NCM (with respect to total solid added).

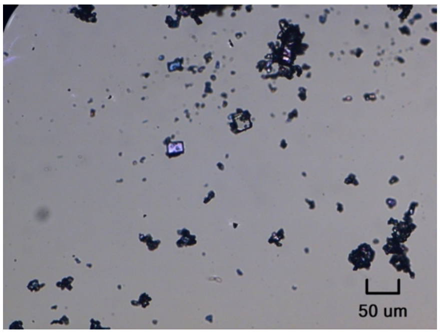

Supplementary Figure 18. Optical microscopy image of crystallites obtained from slurry in isopropanol doped with 20wt.% NCM [initial concentration added to the solution – corresponding to 16% NCM in the crystal – BZM form III solid solution].

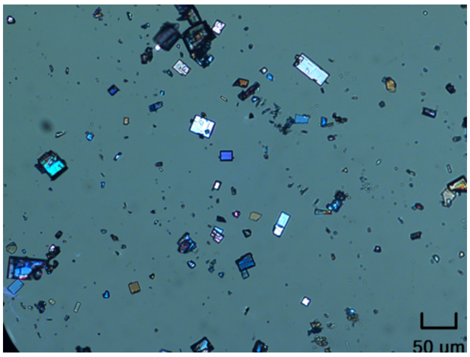

Supplementary Figure 19. Optical microscopy image of crystals obtained from crash cooling crystallisation doped with 20wt.% NCM [initial concentration added to solution – corresponding to 12.9% NCM in the crystal – BZM form III solid solution].

Supplementary References

1. Kobayashi, K., Sato, A., Sakamoto, S. & Yamaguchi, K. Solvent-Induced Polymorphism of Three-Dimensional Hydrogen-Bonded Networks of Hexakis(4-carbamoylphenyl)benzene. *J. Am. Chem. Soc.* **125**, 3035–3045 (2003).
2. Thun, J., Seyfarth, L., Senker, J., Dinnebier, R. E. & Breu, J. Polymorphism in Benzamide: Solving a 175-Year-Old Riddle. *Angew. Chemie Int. Ed.* **46**, 6729–6731 (2007).
3. Kresse, G. & Hafner, J. Ab initio molecular dynamics for liquid metals. *Phys. Rev. B* **47**, 558–561 (1993).
4. Kresse, G. & Hafner, J. Ab initio molecular-dynamics simulation of the liquid-metal--amorphous-semiconductor transition in germanium. *Phys. Rev. B* **49**, 14251–14269 (1994).
5. Kresse, G. & Furthmüller, J. Efficiency of ab-initio total energy calculations for metals and semiconductors using a plane-wave basis set. *Comput. Mater. Sci.* **6**, 15–50 (1996).
6. Kresse, G. & Furthmüller, J. Efficient iterative schemes for ab initio total-energy calculations using a plane-wave basis set. *Phys. Rev. B* **54**, 11169–11186 (1996).
7. Perdew, J. P., Burke, K. & Ernzerhof, M. Generalized Gradient Approximation Made Simple. *Phys. Rev. Lett.* **77**, 3865–3868 (1996).
8. Blöchl, P. E. Projector augmented-wave method. *Phys. Rev. B* **50**, 17953–17979 (1994).
9. Kresse, G. & Joubert, D. From ultrasoft pseudopotentials to the projector augmented-wave method. *Phys. Rev. B* **59**, 1758–1775 (1999).
10. Grimme, S. Semiempirical GGA-type density functional constructed with a long-range dispersion correction. *J. Comput. Chem.* **27**, 1787–1799 (2006).
11. Monkhorst, H. J. & Pack, J. D. Special points for Brillouin-zone integrations. *Phys. Rev. B* **13**, 5188–5192 (1976).
12. Ectors, P. & Zahn, D. Analysis of the molecular interactions governing the polymorphism of benzamide – a guide to syntheses? *Phys. Chem. Chem. Phys.* **15**, 9219–9222 (2013).
13. Ectors, P., Ectors, D. & Zahn, D. Structure and interactions in benzamide molecular crystals. *Mol. Simul.* **39**, 1079–1083 (2013).
14. MestRenova version 14.1.0–24037, Mestrelab Research S.L.,Santiago de Compostela (Spain).
15. Fábián, L. *et al.* Cocrystals of Fenamic Acids with Nicotinamide. *Cryst. Growth Des.* **11**, 3522–3528 (2011).

16. Gelbrich, T. & Hursthouse, M. B. A versatile procedure for the identification{,} description and quantification of structural similarity in molecular crystals. *CrystEngComm* **7**, 324–336 (2005).
