# Supplementary material for: Switching polymorph stabilities with impurities provides a thermodynamic route to benzamide form III
Source: Commun Chem. 2021 Mar 17;4:38. doi: 10.1038/s42004-021-00473-7 (PMC9814557; doi:10.1038/s42004-021-00473-7)
Supplement: Supplementary file 2 — Description of Additional Supplementary Files [file 42004_2021_473_MOESM2_ESM.pdf]

Description of Additional Supplementary Files

File name: Supplementary Data 1

Description: Geometry optimized models for the solid solutions of benzamide and nicotinamide.
